# Application of Fe_3_O_4_/SA nanoparticles and carbonized rice straw biochar as an efficient adsorbents for heavy metals: physiological study on alfalfa plant

**DOI:** 10.1186/s12870-026-09053-0

**Published:** 2026-06-09

**Authors:** Ibrahim Zeid, Yasmin Samir Nabeh, Eman Zakaria Ahmed, Amira Mohamed Abd El-Sattar

**Affiliations:** Botany and Microbiology Department, Faculty of Science, Capital (Helwan) University, Cairo, 11790 Egypt

**Keywords:** Amylase, Antioxidant compounds, Cadmium, Carbonized rice straw, Fe_3_O_4_/SA nanoparticles, Lead, Nickel, Protease

## Abstract

**Background:**

The extensive use of heavy metals in industrial and agricultural fields increases the risk of heavy metals ions accumulation in various plant parts above the maximum permissible limits accepted by FAO and WHO. The objective of this study was to purify irrigation water contaminated with heavy metals to decrease their toxicity on *Medicago sativa* L. by using Fe_3_O_4_/Salicylic acid (Fe_3_O_4_/SA) nanoparticles or carbonized rice straw biochar (C.R.S) as low-cost sorbents.

**Results:**

Characterization of synthesized nanoparticles performed using FT-IR, Zeta sizer, Zeta potential analysis, and UV Vis spectroscopy. Fe_3_O_4_/SA nanoparticles with an average size of 365 nm and a Zeta potential of +ve 18.9 mv were recorded. The adsorbents demonstrated significant capacity for purifying contaminated water with lead, cadmium, and nickel. A mixture of the three heavy metals was prepared at two levels, low and high concentrations. At low concentrations, the highest removal efficiency was achieved for Lead by 97.88%, Cadmium by 95.88% using Fe_3_O_4_/SA nanoparticles and 93.93% for Lead using C.R.S. While Nickel was the least effectively removed of the three heavy metals, reaching an efficiency of 79.51% with Fe_3_O_4_/SA nanoparticles. The use of purified water in irrigation was tested on *Medicago sativa* L.(alfalfa) during early seedling stage. Results showed that heavy metal stress significantly decreased germination percentage by 34%, growth parameters (plumule and radicle length, fresh and dry weight, seedling vigor index, MTI), total soluble proteins and sugars, and the activity of hydrolytic enzymes. At high concentration of heavy metals there was a considerable increase in proline, total phenolics and flavonoids, lipid peroxidation and electrolyte leakage compared to the control. The application of both adsorbents (2 g/L) significantly reduced the inhibitory effects of heavy metals and improved physiological and biochemical indicators through recovery and increase in the activity of hydrolytic enzymes. This led to a notable increase in the germination percentage that reached 99% compared to control 92%, plumule length recovered to 6.5 cm at L. C + C.R.S and radicle length to 3.5 cm at L. C + Fe_3_O_4_/SA nanoparticles, total soluble sugars, and protein content increased. Conversely, there was a significant decrease in the stress indicators, proline to 4.7 mg g⁻¹ f.wt, lipid peroxidation dropped to 28.1 µM g⁻¹ f.wt and antioxidants compounds content also decreased at the treatment L. C + C.R.S.

**Conclusion:**

The research highlighted that both adsorbents are effective, eco-friendly, and low-cost techniques for removing heavy metals and in turn enhancing water quality which was reflected on alfalfa plant growth irrigated with purified water.

**Graphical Abstract:**

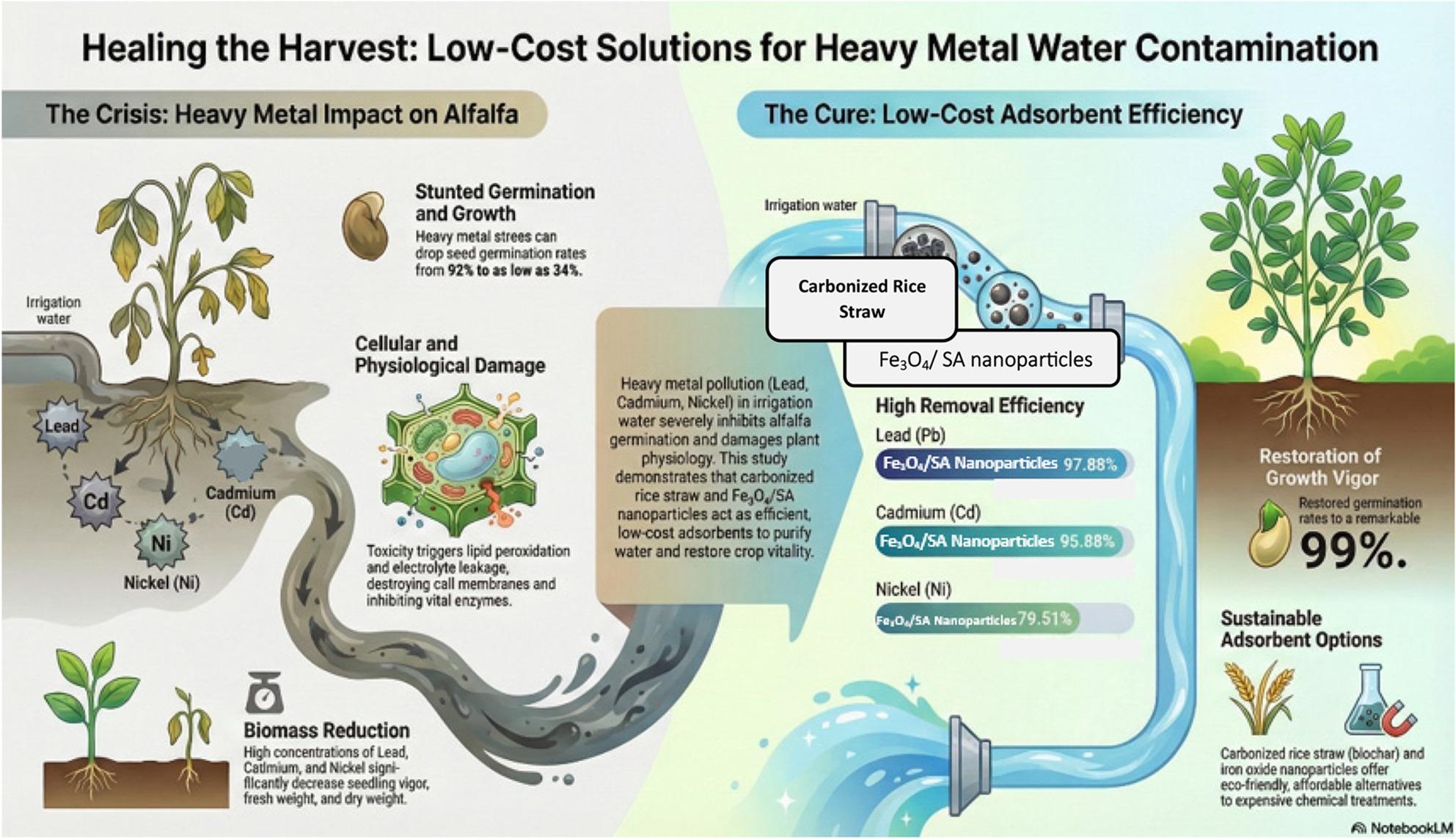

## Introduction

*Medicago sativa* L. is a perennial leguminous forage crop that is widely planted because of its high productivity, adaptability, positive quality traits, and versatility. Although its primary usage is still as animal feed, its high protein content is making it increasingly attractive for human consumption [1]. According to Mouttet et al. [2], there were about 30 million hectares of alfalfa growing worldwide, with 41% in North America, 25% in Europe, 23% in South America, 8% in Asia, and 3% in Africa and Oceania. In addition to green manure and cover crops, it serves for purposes such as grazing, hay, and silage. With a dry matter crude protein level of 26% to 30%, alfalfa leaves are a major source of protein, however the stems have a much lower dry matter crude protein value of 10–12% [3]. Alfalfa is considered valuable for its deep root system, which not only stops soil erosion but also boosts soil fertility with nitrogen. It is native to warmer temperate climates [4]. Human health is under grave danger due to the heavy metal issue. These metals are also carcinogenic and poisonous and severely harm ecosystems [5]. Understanding soil water balance is crucial for optimizing irrigation schedules in arid regions, which directly impacts the mobilization and transport of heavy metals in agricultural systems [6]. A class of naturally occurring elements originating from the earth’s crust are known as heavy metals (HM). However, human activities such as mining, agriculture, and industrial discharges can introduce more heavy metals into the environment [7]. According to Jacob et al. [8], the environment is recognized to be seriously threatened by dangerous heavy metals such manganese (Mn), aluminum (Al), nickel (Ni), zinc (Zn), copper (Cu), chromium (Cr), lead (Pb), arsenic (As), cadmium (Cd), manganese (Mn), and copper (Cu). According to Wang et al. [9], cadmium (Cd) is a hazardous heavy metal that discharged into the environment via a variety of manufactured and natural sources, including the burning of fossil fuels, metal mining and refining, and the manufacture of batteries and pigments. It has detrimental effects on the kidneys and bones. As stated by Musstjab et al. [10], lead is a toxic heavy metal that is used extensively in a variety of industries, and steam-electric power plants. The use of liquid and solid fuels, as well as municipal and industrial waste, can all contribute to nickel pollution in the environment [11].

Chemical precipitation, solvent extraction, chemical oxidation, ion exchange, membrane separation, reverse osmosis, and electro-dialysis are the traditional methods used to address heavy metal contamination. However, these techniques were unpopular due to several drawbacks, including their higher cost, high energy consumption, and frequent production of hazardous byproducts. Because of its higher removal efficacy for heavy metal ion concentration from wastewater, the adsorption process has thus been investigated as one of the most economical methods [12]. Large-scale natural resources or specific waste products from commercial or agricultural processes may be promising as low-cost sorbents. Because of their low cost, these materials can be disposed of without costly regeneration when their useful lives are ending. Agricultural by-products are a suitable source of raw materials for natural sorbents due to their availability and quantity [13]. According to earlier studies, there is an increasing interest in finding a range of materials as inexpensive adsorbents, such as rice straw [14], carbonized rice straw [15], metal oxide-based synthetic nanoparticles like zinc oxide [16], and iron oxide salicylic acid nanoparticles [17].

A sustainable substitute for commercial wastewater treatment systems, which are typically costly and chemical-intensive, is biochar, a low-cost and environmentally friendly adsorbent [18, 19]. Biochar is a carbon-rich substance created by heating biomass, such as wood, manure, or leaves, to 400–700 °C in a closed container with little or no oxygen present [20]. Condensed carbonaceous matter, a unique pore structure, active functional groups, facile modification, and stable chemical characteristics are some of the advantages of biochar over other adsorbents [21]. Biochar can be used to effectively remove water pollutants, recover nutrients, control urban runoff, and lessen industrial pollution [22]. According to El-Sattar and Shedeed [23], biochar has been shown to improve plant growth and productivity even under abiotic stress.

The high surface area to volume ratio of synthetic nanoparticles (Fe_3_O_4_/salicylic acid nanocomposite) from nano-adsorbents has attracted a lot of attention for heavy metal adsorption because it offers many reactive sites for HM adhesion compared to the same material at higher dimensions [24]. For HMs found in waste effluents, this makes nanomaterials superior adsorbents. The interaction between adsorbents and nanoparticles, which is dependent on their physicochemical characteristics, is the primary focus of the adsorption approach [25]. Particle size, composition, and surface charge all have an impact on nanoparticle aggregation. The inability of nanoparticles having non-magnetic characteristics to separate from the aqueous solution limits their use in water purification [26]. A magnet field can be used to separate heavy metals from the aqueous solution, and their enormous surface area and size and shape-dependent catalytic capabilities make them advantageous [27]. The eco-friendly magnetic adsorbent Fe_3_O_4_/salicylic acid nanocomposite was prepared using a simple one-pot co-precipitation technique, and it was utilized as a magnetic solid-phase extraction agent to separate Cd ions from artificial solutions [28]. Salicylic acid (SA) plays a significant role in many metabolic processes. In plants exposed to stress conditions, it controls ionic uptake, photosynthesis, antioxidant activity, redox and osmotic hemostasis, and the synthesis of secondary metabolites [29]. Iron oxide nanoparticles reduced solute leakage and increased metabolic activity of soluble sugar and amino acids, which regulated seed viability. Additionally, the administration of nanoparticles enhanced defense mechanisms and decreased oxidative damage by inducing peroxidase and dehydrogenase activities [30]. The application of both SA and iron nanoparticles improved all growth-related parameters and increased the pigment content, relative water content, membrane stability index, and iron and potassium contents of the mature plants [31].

The aim of this search is to highlight the synthesis of inexpensive adsorbents, such as carbonized rice straw or Fe_3_O_4_/SA nanoparticles, and their efficiency in heavy metals adsorption from polluted irrigation water.

## Materials and methods

### Preparation of adsorbents

#### Carbonized rice straw

Rice straw was filtered and dried at 100 °C after being repeatedly cleaned with deionized water to eliminate all dirt particles in their natural size. In a muffle furnace, the rice straw was heated to 300 to 400 °C for three hours while enclosed in a porcelain cup under extraordinarily little oxygen. Therefore, the slow pyrolysis method was applied in this study. A mortar and pestle were used to grind Carbonized rice straw materials, which were subsequently sieved through a 0.5-mm sieve according to [32]. The physical and chemical properties of the sample produced were investigated. The EC and pH of the carbonized rice straw were measured using a conductivity meter and a pH meter with gloss electrodes using a suspension (1:10 w/v) and standard buffer solutions. The mineral ions content was measured using Microwave Plasma Atomic Emission Spectrometer (MPAES, Agilent, Santa Clara, CA 95051, United States) at the Ecology Laboratory, Faculty of Science, Helwan University.

#### Iron oxide salicylic acid nanoparticles

Synthesis of the Fe_3_O_4_/ SA nanoparticles followed recommendations provided in the literature [33]. Deionized water 250 mL was used to dissolve 1.25 g of FeSO_4_.7H_2_O and 2 g of FeCl_3_.6H_2_O. A 250 mL solution containing 0.5 g of SA and 10 g of KOH at 50 °C was then used to precipitate the mixture. The solution obtained was heated to 50 °C for 30 min. The final product was removed using a magnet once it had cooled to room temperature and then rinsed three times with deionized water to get rid of extra salicylate and KOH. Finally, magnetic Fe_3_O_4_/SA nanoparticles were obtained by drying the product.

#### Characterization of Fe_3_O_4_/SA nanoparticles

**Fig. 1:**
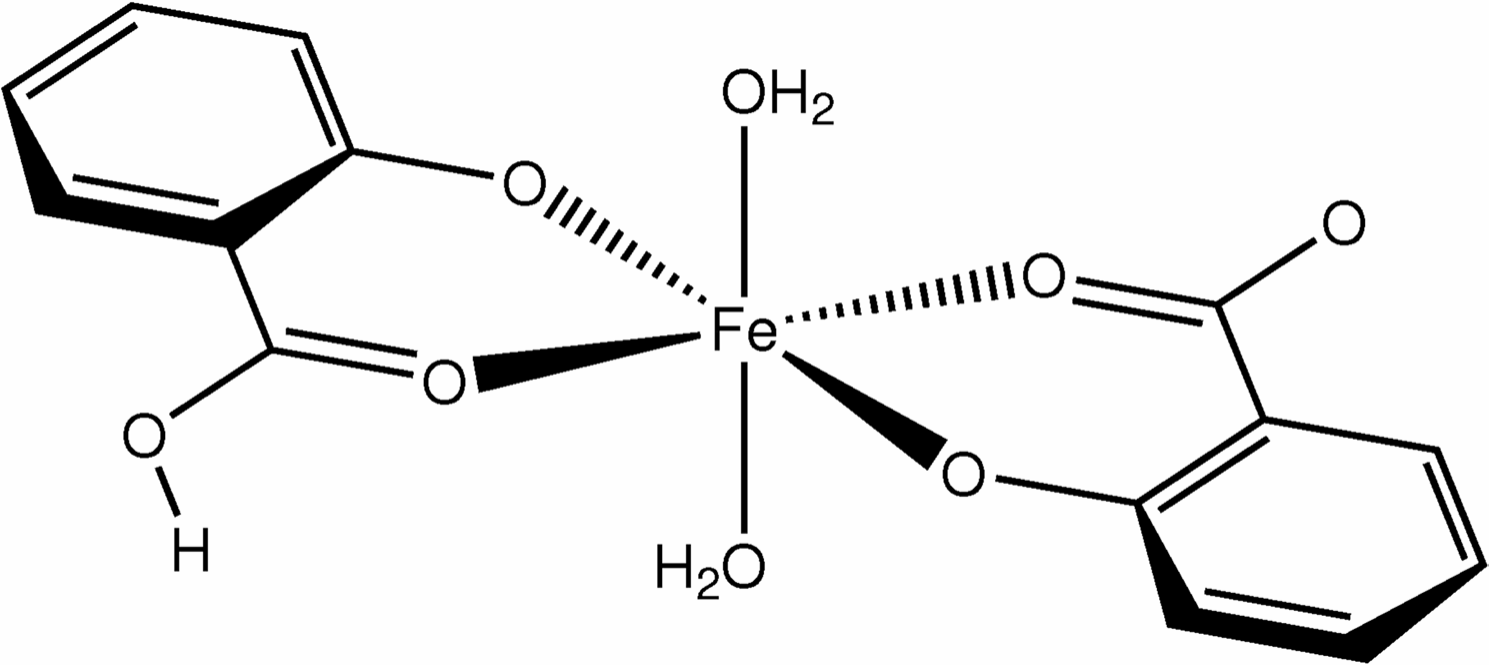
Molecular structure of iron (III) Salicylate compound

Several methods were used to confirm the formation of iron oxide salicylic acid nanoparticles and determine their size, structure, and stability. Using a T80 + UV/VIS Spectrometer made by PG Instruments Ltd. in the Bacteriology lab of the Faculty of Science at Helwan University in Egypt, the generated Fe_3_O_4_/SA nanoparticles absorption peak was described throughout a wavelength range of 200–800 nm. Solid powder samples were used to perform the Fourier Transform Infrared (FTIR) spectra of Fe_3_O_4_/SA nanoparticles. The spectra were obtained at a resolution of 2 cm⁻¹ within the 400–4000 cm⁻¹ wave number range. Using a Zeta sizer Nano ZS particle size analyzer from Malvern Instruments, UK, the zeta potential and particle size were determined at the Nanotechnology Research Center at Helwan University (Capital University) in Egypt. The molecular structure of iron (III) Salicylate compound (Fig. 1) [34].

### Preliminary experiments

**Fig. 2:**
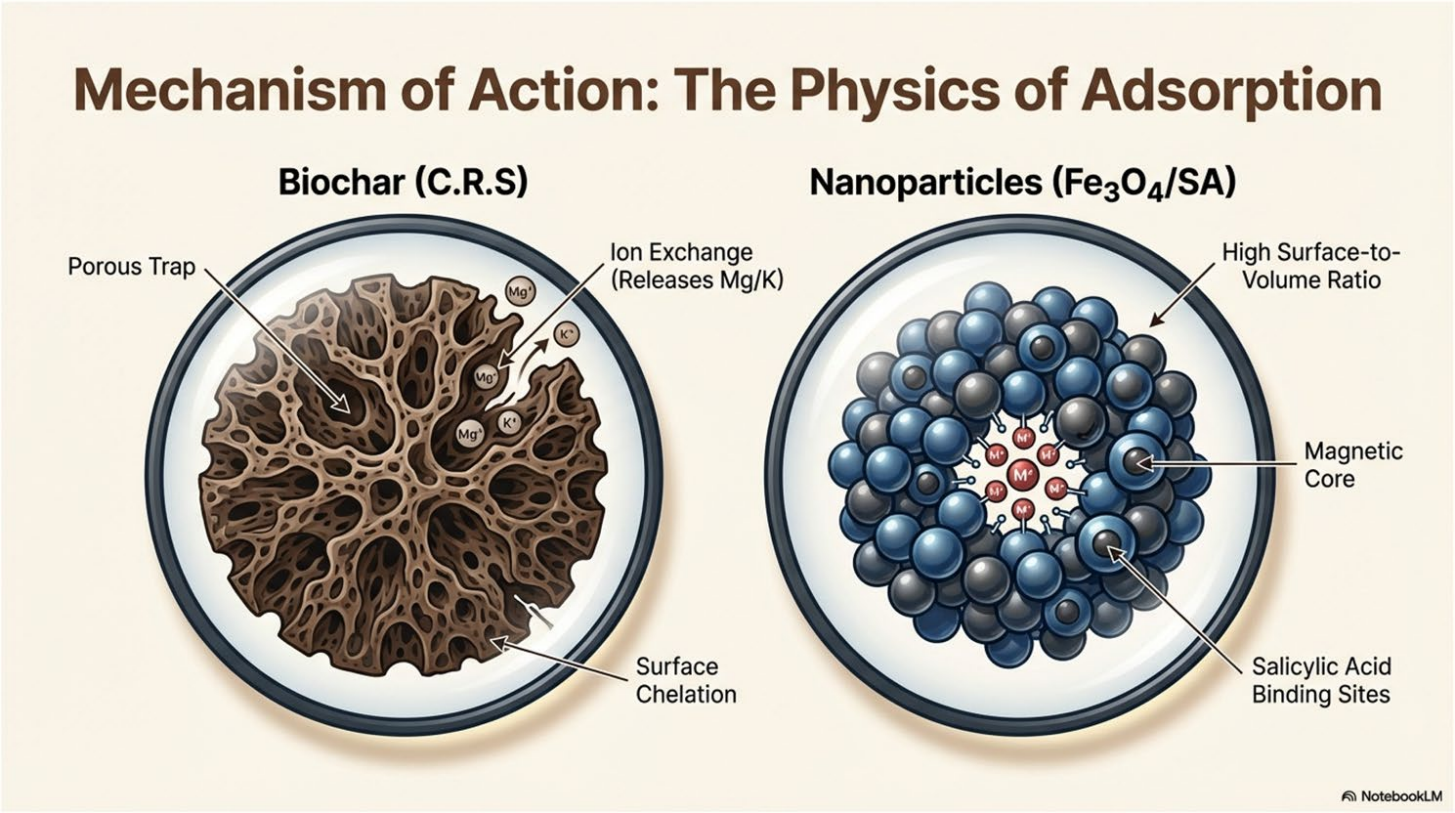
Mechanism of adsorption process of C.R.S and Fe_3_O_4_/SA nanoparticles designed by Notebook LM

Different concentrations of lead acetate (500, 1000, 1500 ppm), cadmium nitrate (25, 50, 75, 100 ppm), and nickel chloride (100, 200, 300, 350 ppm) were prepared to assess the toxicity of heavy metals on alfalfa seed germination. Four replicates for each concentration, each containing a fixed number of sterilized seeds (25 seeds), were placed in a sterile petri dish with a single Whatman No. 1 filter paper. The seeds were allowed to germinate in 10 ml solution; distilled water used for control. After 4 days of incubation at 25 °C, germination percent was calculated. According to the preliminary experiment results, two heavy metals solutions (low and high) were prepared for the main experiment. Low-concentration (L. C) solution was prepared by mixing equal volumes of 500 ppm Pb, 50 ppm Cd, and 100 ppm Ni stock salt solutions, resulting in a final concentration of the three metals in the mixture 20.29 ppm Pb, 13.85 ppm Cd, and 54.09 ppm Ni calculated based on metals atomic number. High concentration (H. C) was prepared by mixing equal volumes of 1000 ppm Pb, 75 ppm Cd, and 200 ppm Ni stock solutions resulting in final concentrations of 58.41 ppm Pb, 22.68 ppm Cd, and 98.23 ppm Ni calculated based on metal atomic number. The Fe_3_O_4_/SA nanoparticles and carbonized rice straw (2 g/L) were mixed with both the low concentration (L. C) and the high concentration (H. C) of the heavy metals for 24 h at room temperature and pH (7) to achieve the highest adsorption equilibrium state then the solutions were filtered using Whatman filter paper No. 1. Microwave Plasma Atomic Emission Spectrometer (MPAES, Agilent, Santa Clara, CA 95051, United States) was used to measure final Pb, Cd and Ni concentrations in filtrate to assess the effectiveness of adsorbents for the removal of heavy metals. The suggested mechanism of adsorption process of C.R.S and Fe_3_O_4_/SA nanoparticles shown in (Fig. 2).

### Germination experiment

Alfalfa seeds (Ramah 1) were provided from the Field Crops Research Institute’s Legumes Crops Department at the Agricultural Research Center, Giza, Egypt. Sodium hypochlorite solution (2.5%) was used to sterilize the carefully selected alfalfa seeds for three minutes, and they were then thoroughly rinsed with distilled water several times. Four replicates for each treatment, each containing a fixed number of seeds (25 seeds), were placed in a sterile petri dish with a single Whatman No. 1 filter paper. The seeds were allowed to germinate in 10 ml solution, distilled water (Control), low concentrations of heavy metals (L. C), high concentrations of heavy metals (H. C), L. C treated with C.R.S (L. C + C.R.S), L. C treated with Fe_3_O_4_/SA nanoparticles(L. C+ Fe_3_O_4_/SA), H. C treated with C.R.S (H. C + C.R.S) and H. C treated with Fe_3_O_4_/SA nanoparticles (H. C+ Fe_3_O_4_/SA). The petri dishes were incubated at 25 °C. When the plumule and radicle reached a length of 2 mm, the seed was deemed to have germinated [35]. Data on germination %, biochemical assays, and growth metrics (seedling fresh weight and dry weights (mg) and seedling vigor index) were collected at early seedling stage (seven days later). To calculate their dry weight, seedlings were dried for 48 h at 65 °C in an oven. According to Iqbal and Rahmati [36], the seedling vigor index was calculated according to Chou and Lin’s [37].

$$\text{Seedling Vigor index}\;\left(\mathrm{SVI}\right)=\left(\mathrm{RL}+\mathrm{PL}\right)\times\mathrm{GP}$$

Formula was used to calculate the inhibitory (%) of radical and plumule seedlings.

$$\:\mathrm{I}\mathrm{n}\mathrm{h}\mathrm{i}\mathrm{b}\mathrm{i}\mathrm{t}\mathrm{i}\mathrm{o}\mathrm{n}\:\left(\%\right)\mathrm{o}\mathrm{f}\:\mathrm{r}\mathrm{a}\mathrm{d}\mathrm{i}\mathrm{c}\mathrm{l}\mathrm{e}\:\mathrm{g}\mathrm{r}\mathrm{o}\mathrm{w}\mathrm{t}\mathrm{h}\:=\frac{\mathrm{R}\mathrm{a}\mathrm{d}\mathrm{i}\mathrm{c}\mathrm{l}\mathrm{e}\:\mathrm{l}\mathrm{e}\mathrm{n}\mathrm{g}\mathrm{t}\mathrm{h}\:\mathrm{o}\mathrm{f}\:\mathrm{c}\mathrm{o}\mathrm{n}\mathrm{t}\mathrm{r}\mathrm{o}\mathrm{l}-\mathrm{R}\mathrm{a}\mathrm{d}\mathrm{i}\mathrm{c}\mathrm{a}\mathrm{l}\:\mathrm{l}\mathrm{e}\mathrm{n}\mathrm{g}\mathrm{t}\mathrm{h}\:\mathrm{o}\mathrm{f}\:\mathrm{t}\mathrm{r}\mathrm{e}\mathrm{a}\mathrm{t}\mathrm{m}\mathrm{e}\mathrm{n}\mathrm{t}}{\mathrm{R}\mathrm{a}\mathrm{d}\mathrm{i}\mathrm{c}\mathrm{l}\mathrm{e}\:\mathrm{l}\mathrm{e}\mathrm{n}\mathrm{g}\mathrm{t}\mathrm{h}\:\mathrm{o}\mathrm{f}\:\mathrm{c}\mathrm{o}\mathrm{n}\mathrm{t}\mathrm{r}\mathrm{o}\mathrm{l}}\times\:100$$

$$\:\mathrm{I}\mathrm{n}\mathrm{h}\mathrm{i}\mathrm{b}\mathrm{i}\mathrm{t}\mathrm{i}\mathrm{o}\mathrm{n}\:\left(\%\right)\mathrm{o}\mathrm{f}\:\mathrm{p}\mathrm{l}\mathrm{u}\mathrm{m}\mathrm{u}\mathrm{l}\mathrm{e}\:\mathrm{g}\mathrm{r}\mathrm{o}\mathrm{w}\mathrm{t}\mathrm{h}\:=\frac{\mathrm{P}\mathrm{l}\mathrm{u}\mathrm{m}\mathrm{u}\mathrm{l}\mathrm{e}\:\mathrm{l}\mathrm{e}\mathrm{n}\mathrm{g}\mathrm{t}\mathrm{h}\:\mathrm{o}\mathrm{f}\:\mathrm{c}\mathrm{o}\mathrm{n}\mathrm{t}\mathrm{r}\mathrm{o}\mathrm{l}-\mathrm{P}\mathrm{l}\mathrm{u}\mathrm{m}\mathrm{u}\mathrm{l}\mathrm{e}\:\mathrm{l}\mathrm{e}\mathrm{n}\mathrm{g}\mathrm{t}\mathrm{h}\:\mathrm{o}\mathrm{f}\:\mathrm{t}\mathrm{r}\mathrm{e}\mathrm{a}\mathrm{t}\mathrm{m}\mathrm{e}\mathrm{n}\mathrm{t}}{\mathrm{P}\mathrm{l}\mathrm{u}\mathrm{m}\mathrm{u}\mathrm{l}\mathrm{e}\:\mathrm{l}\mathrm{e}\mathrm{n}\mathrm{g}\mathrm{t}\mathrm{h}\:\mathrm{o}\mathrm{f}\:\mathrm{c}\mathrm{o}\mathrm{n}\mathrm{t}\mathrm{r}\mathrm{o}\mathrm{l}}\times\:100$$

Using the formula developed by Turner and Marshal [38], the metal tolerance index (MTI) was computed by dividing the radicle length at each treatment by that found in the control.

$$\:\mathrm{M}\mathrm{e}\mathrm{t}\mathrm{a}\mathrm{l}\:\mathrm{T}\mathrm{o}\mathrm{l}\mathrm{e}\mathrm{r}\mathrm{a}\mathrm{n}\mathrm{c}\mathrm{e}\:\mathrm{I}\mathrm{n}\mathrm{d}\mathrm{e}\mathrm{x}\:\:\left(\mathrm{M}\mathrm{T}\mathrm{I}\right)=\frac{\mathrm{R}\mathrm{a}\mathrm{d}\mathrm{i}\mathrm{c}\mathrm{l}\mathrm{e}\:\mathrm{l}\mathrm{e}\mathrm{n}\mathrm{g}\mathrm{t}\mathrm{h}\:\mathrm{o}\mathrm{f}\:\mathrm{s}\mathrm{t}\mathrm{r}\mathrm{e}\mathrm{s}\mathrm{s}\mathrm{e}\mathrm{d}\:\mathrm{s}\mathrm{e}\mathrm{e}\mathrm{d}\mathrm{s}}{\mathrm{R}\mathrm{a}\mathrm{d}\mathrm{i}\mathrm{c}\mathrm{l}\mathrm{e}\:\mathrm{l}\mathrm{e}\mathrm{n}\mathrm{g}\mathrm{t}\mathrm{h}\:\mathrm{o}\mathrm{f}\:\mathrm{c}\mathrm{o}\mathrm{n}\mathrm{t}\mathrm{r}\mathrm{o}\mathrm{l}\:\mathrm{s}\mathrm{e}\mathrm{e}\mathrm{d}\mathrm{s}}\times\:100$$

### Biochemical analyses

#### α- Amylase enzyme assay

The quantitative assessment of α-amylase activity was conducted using Miller’s method [39] 3,5-dinitrosalicylic acid technique. 1 mL of the enzyme extract was mixed with 1 mL of 1% soluble starch that had been dissolved in pH 5.6 sodium acetate buffer. The mixture was incubated at 40 °C for 15 min, and then 2 ml of 3,5-dinitrosalicylic acid were added to each tube and incubated for 5 min in a boiling water bath. A UV-vis spectrophotometer set to 540 nm was used to measure the generated color.

#### Protease enzyme

The assaying mixture for protease consisted of 1 ml of casein (1%) in phosphate buffer (pH 7.5) and 1 ml of enzyme extract. After an hour of incubation at 37 °C, 2 ml of 10% trichloroacetic acid was added to terminate the reaction. The mixture was then centrifuged at 4000 rpm for 20 min at 4 °C. The content of soluble peptides in the supernatant was determined [40].

#### Total soluble proteins

Five milliliters of freshly mixed (1:1 v/v) solutions of 2% sodium carbonate in 0.4% sodium hydroxide and 0.5% copper sulphate in 1% sodium potassium tartrate were added to 1 ml sample extract to assess the total soluble proteins using the Lowry et al. [40] technique. After ten minutes of standing, 0.5 ml of Folin was added to the mixture. The optical density of the combination at 750 nm was measured after 30 min.

#### Total soluble sugars

The known weight of germinated seeds was grinded in five milliliters of 70% ethanol. After centrifugation the supernatant was completed to a known volume by distilled water. After mixing one milliliter of the extract with two milliliters of anthrone reagent, the mixture was placed in a boiling water bath for three minutes [41]. The produced color was measured spectrophotometry at 620 nm after cooling.

#### Hydrogen peroxide

Hydrogen peroxide content was determined according to Velikova et al. [42]. In an ice bath, 0.5 g of fresh plant material was homogenized with 5 mL of trichloroacetic acid (TCA), 0.1% (w/v). For 15 min, the homogenate was centrifuged at 12,000 rpm. The supernatant was mixed with 1 mL of 1 M potassium iodide (KI) and 0.5 mL of 10 mM potassium phosphate buffer (pH 7.0) before adding to the mixture. The supernatant’s absorbance was determined at 390 nm.

#### Lipid peroxidation

The weight of 0.2 g sprouts was homogenized in 3 ml of 50 mM phosphate buffer at a pH of 7.0 using Heath and Packer [43] technique to measure the amount of lipid peroxidation. After 20 min of centrifugation, a 1 ml aliquot of the supernatant was mixed with 2 ml of 0.5% 2-thiobarbituric acid in 20% trichloroacetic acid. After being heated in the water bath for 35 min to 96 °C, the mixture was cooled in an ice bath. After a 15-minute centrifugation interval, the absorbance of the supernatant was measured at a wavelength 532 nm and 600 nm.

#### The electrolyte leakage

Hamed et al. [44] approach was used to measure electrolyte leakage. In glass test tubes, 300 mg of plant samples and 15 ml of ultra-pure water were combined. These test tubes were incubated for three hours at 25 °C in a water bath before their basic electrical conductivity was measured using an EC meter. After 20 min of heating to 96 °C, the plant samples were cooled, and the solution’s EC was once again measured. The electrolyte leakage % was determined using the following formula:

$$\text{Electrolyte leakage}=\;\left(\text{EC elementary}/\;\text{EC final}\right)\times100$$

#### Proline content

Following the method outlined by Bates et al. [41] for measuring proline, 0.5 g of germinated seeds were homogenized in 10 ml of 3% aqueous sulfosalicylic acid, and the homogenate was then filtered through Whatman No. 1 paper. Two milliliters of glacial acetic acid and acid ninhydrin were added to a two-milliliter aliquot of filtrate, which was heated to 100 °C for an hour. The reaction was halted for fifteen minutes on ice. After that, 4 mL of toluene was added to the reaction mixture, and it was vortexed for 20 s. A spectrophotometer was used to measure the absorbance of the upper phase at 520 nm.

#### Total phenolics content

Total phenolics content was measured according to Chun et al. [45]. Folin-Ciocalteu reagent (2.5 ml) was mixed with 0.5 ml plant extract then incubated at 25 °C for 8 min. After eight minutes, this solution was combined with two milliliters of a 7.5% sodium carbonate solution. After 15 min, the total phenolic content was determined at a wavelength of 765 nm.

#### Total flavonoids

The total flavonoids were calculated using quercetin as a standard, which was developed by [46], in accordance with the aluminum chloride method published by Zhishen et al. [47]. One milliliter of extract was added to 0.3 ml of 10% AlCl_3_ then incubated for 5 min at room temperature before adding 0.30 ml of 5% NaNO_2_. Following a 5-minute waiting time, 10 ml of distilled water and 2 ml of 1 M NaOH were added to the mixture. The absorbance was measured at wavelength of 510 nm.

### Statistical analyses

IBM SPSS Statistics Version 21 Software was used in results analysis using a one-way ANOVA. Dunkun’s HSD test was used to identify the significant differences at α = 0.05.

## Results

### Characterization of carbonized rice straw

The physical and chemical properties *of* the sample produced (Table 1), indicated that pH was slightly alkaline, EC was 1.62 (ds m^-1^), a high carbon content 58.8% and contain a high minerals content as, P (10.75%), K (2.43%) and Mg (14.56%).

**Table 1:** The physico-chemical properties of carbonized rice straw

The measured property or element	The value
pH	7.8
EC (ds m^-1^)	1.62
Ash content (%)	41
Carbon content (%)	58.8
H (%)	2.84
Nitrogen (%)	0.73
P (%)	10.75
K (%)	2.43
Mg (%)	14.56
Mn (%)	0.022
Fe (%)	0.59
Cu (%)	0.33

### Characterization of Fe_3_O_4_/SA nanoparticles

**Fig. 3:**
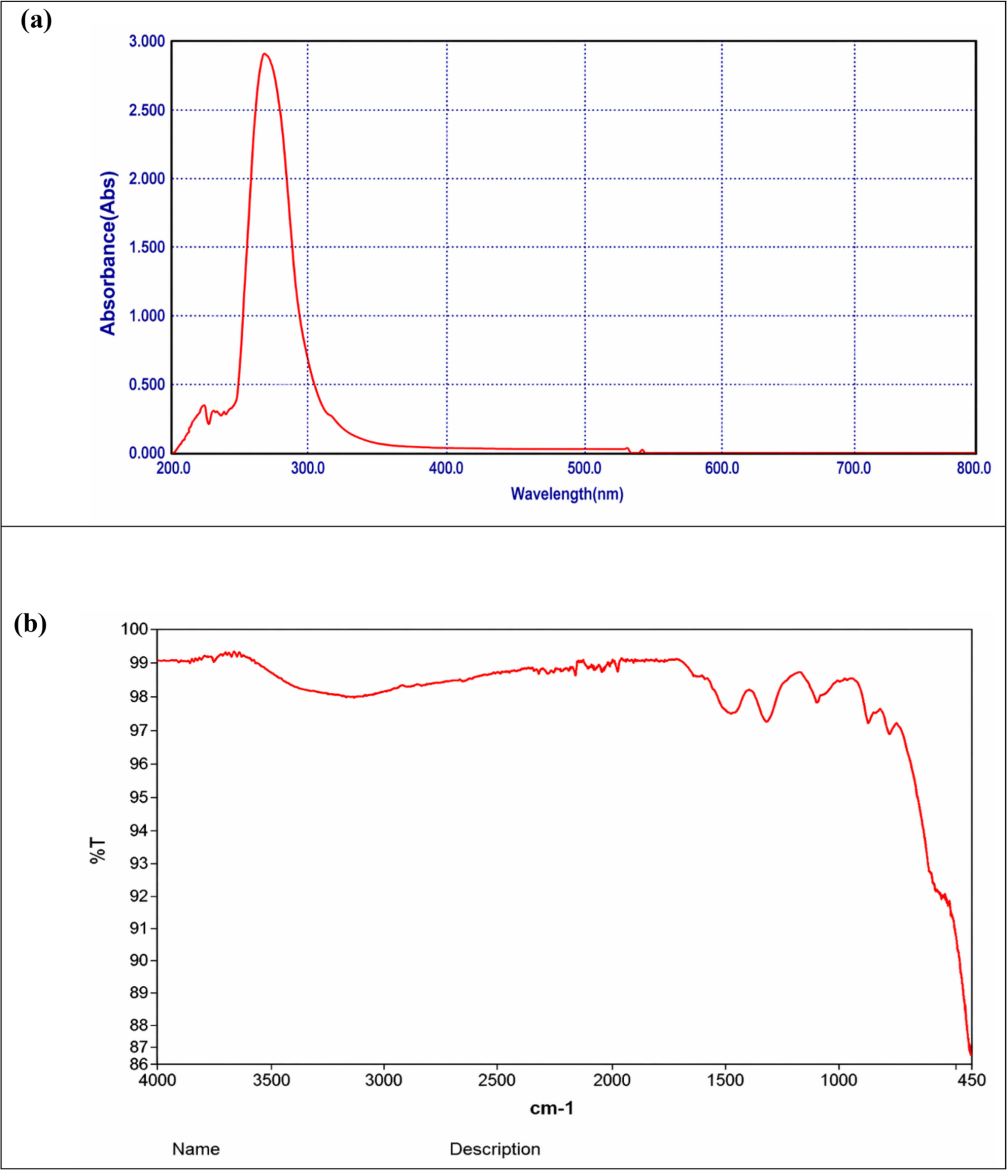
Characterization of synthesized Fe_3_O_4_/SA nanoparticles by (**a**) UV–visible absorbance spectrum analysis (**b**) FT-IR analysis

**Fig. 4:**
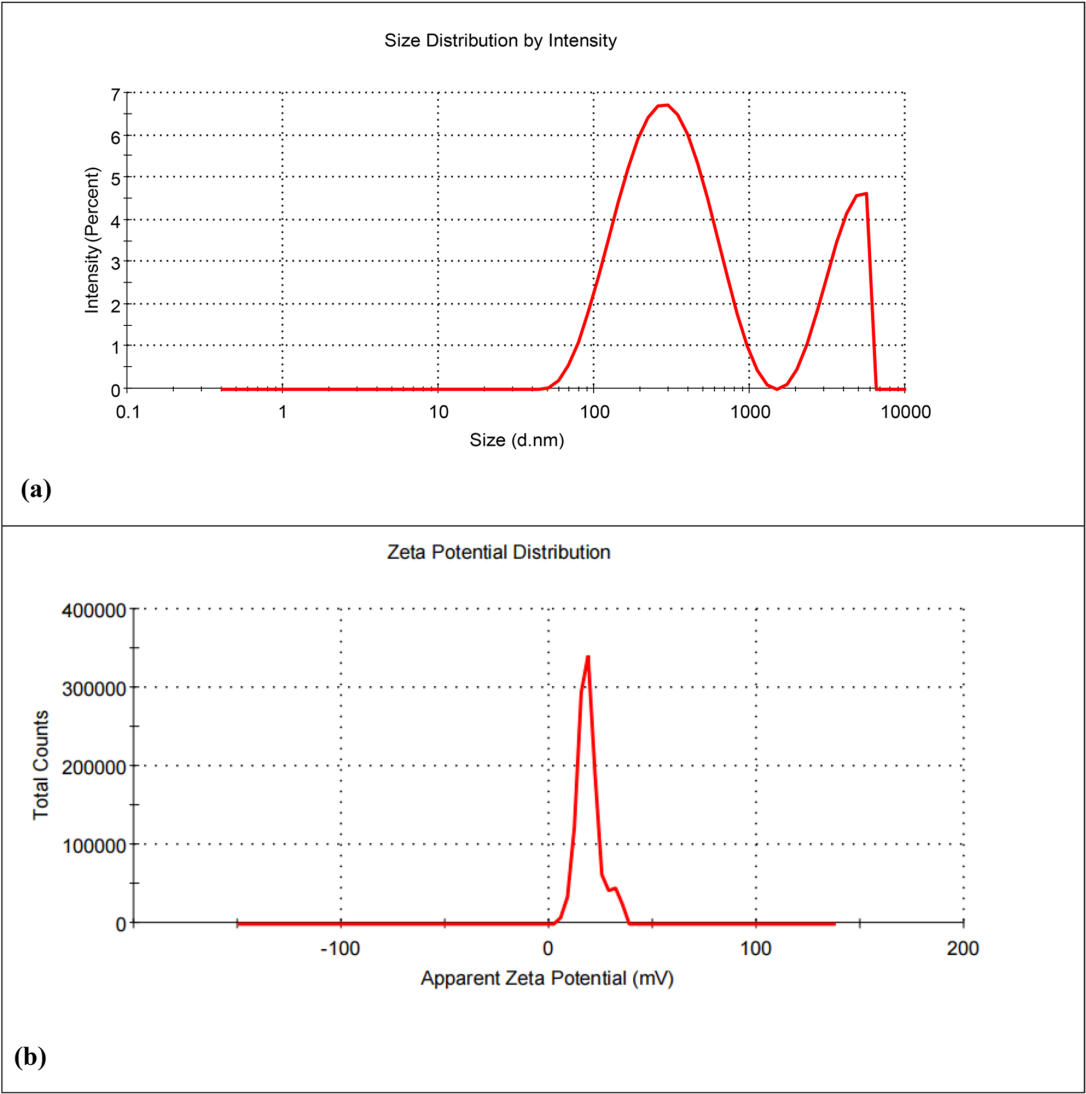
Characterization of synthesized Fe_3_O_4_/SA nanoparticles (**a**) size and (**b**) zeta potential

The synthesis of Fe_3_O_4_/SA nanoparticles was detected between 200 and 800 nm range. UV-Vis’s spectroscopy showed that the nano Fe_3_O_4_/SA solution has a peak at 273 nm (Fig. 3a). Iron oxide / salicylic acid nanoparticles may exhibit a combination of the peaks linked to both iron oxide and salicylic acid in their FTIR spectra, with shifts or variations in strength based on the interaction. The FTIR spectra of Fe_3_O_4_/SA nanoparticles showed various bands with slightly shifting wave numbers; absorption bands were found at wave numbers 3140.16, 1482.96, 1332.90, and 1106.22, 650 cm⁻¹ (Fig. 3b). The hydrodynamic diameter determined by the DLS technique showed a particle size distribution of approximately 365 nm and a Zeta potential of +ve 18.9 mv which indicates that the nanoparticles possess a positive surface charge, this value reflects low to moderate colloidal stability average. (Fig. 4a, b).

### The efficiency of C.R.S or Fe_3_O_4_/SA nanoparticles adsorbents

**Fig. 5:**
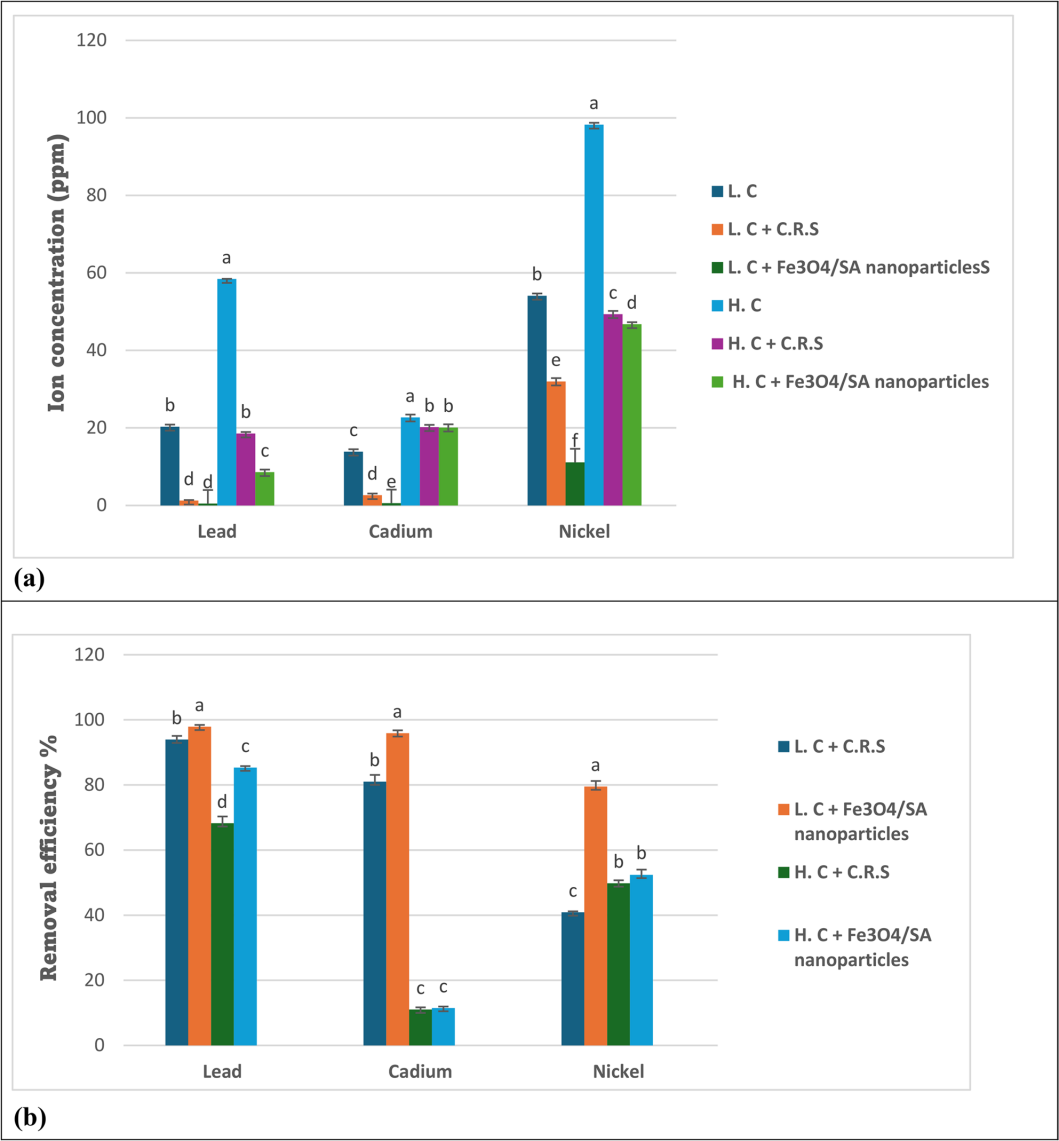
**a** Pb, Cd and Ni ions concentration in low concentrations solution (L.C) used of lead acetate (500 ppm), cadmium nitrate (50 ppm) and nickel chloride (100 ppm), and in a solution high concentrations solution (H.C) used of lead acetate (1000 ppm), cadmium nitrate (75 ppm) and nickel chloride (200 ppm) before and after application of carbonated rice straw and Fe_3_O_4_/SA nanoparticles treatments, (**b**) Removal efficiency % of carbonized rice straw and Fe_3_O_4_/SA nanoparticles in both L.C and H.C. solutions. Different letters (a, b, c, d and e) indicate statistical differences at 5% probability according to Duncan’s test. Error bars are standard errors of the mean

The concentrations of Pd, Cd and Ni ions were represented in (Fig. 5a) before and after application of different adsorbents. Pd was 20.29 and 58.42 ppm in the prepared lead acetate solutions at 500 and 1000 ppm. Cd was 13.85 and 22.6 ppm in the prepared cadmium nitrate solutions at 50 and 75 ppm. Ni was 54.09 and 98.23 ppm in the prepared nickel chloride solutions at 100 and 200 ppm. The treatments C.R.S or Fe_3_O_4_/SA nanoparticles reduced the free ions concentration, and the removal efficiency of each treatment were represented in (Fig. 5b). At low concentrations treated with C.R.S or Fe_3_O_4_/SA, Pd significantly decreased to 1.23 and 0.45 ppm respectively (by removal efficiency percentage 93.93 and 97.88%, respectively), Cd decreased to 2.63 and 0.57 ppm (by removal efficiency percentage 81.01 and 95.88%, respectively), while Ni decreased to 31.97 and 11.08 ppm (by removal efficiency percentage 40.89 and 79.51%, respectively). At high concentrations Pd decreased to 18.54 and 8.56 ppm (by removal efficiency percentage 68.24 and 85.34%, respectively), Cd decreased to 20.18 and 20.07 ppm (by removal efficiency percentage 11.02 and 11.50%, respectively), while Ni recorded 49.33 and 46.75 (by removal efficiency percentage 49.78 and 52.40%, respectively).

### Germination percentage

**Fig. 6:**
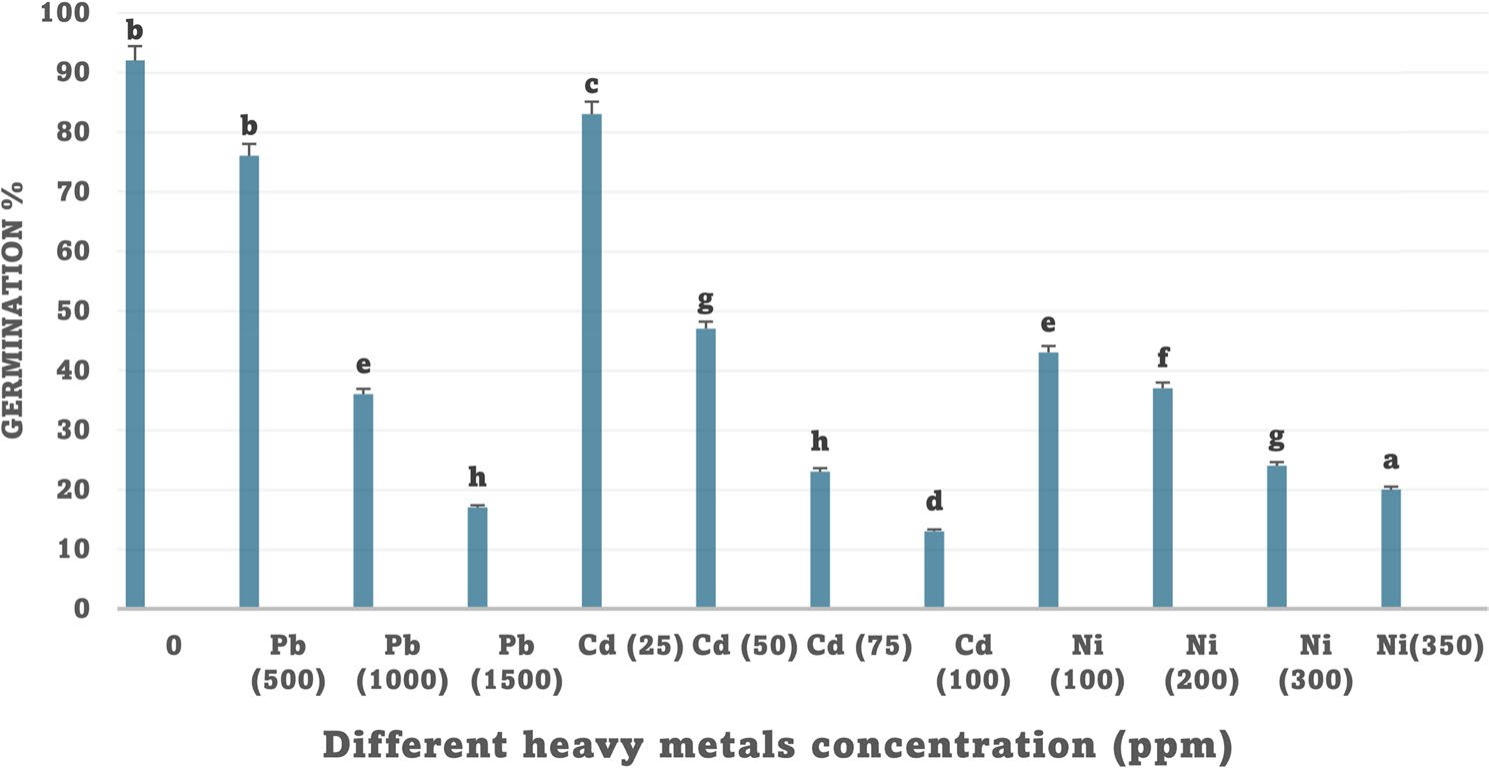
Effect of different concentrations of lead acetate, cadmium nitrate and nickel chloride on seed germination percentage of alfalfa. (4 days old seedlings). Values represent the mean of three replicates. Different letters (a, b, c, d, e, f, g and h) indicate statistical differences at 5% probability according to Duncan’s test. Error bars are standard errors of the mean

In the preliminary experiment, germination percentage of alfalfa seed decreased gradually as heavy metal concentration increased, reaching complete inhibition at high concentrations *of *Pb, (1500 ppm), cd (100 ppm) and Ni, (350 ppm) (Fig. 6).

The mixture of the three heavy metals at both low and high concentrations decreased germination percentage to 65% and 34% respectively. Treatment of low and high heavy metals mixtures with carbonized rice straw decreased heavy metals content in water and in turn alleviated the inhibitory impact, causing increase in germination percentage to 94% and 69% at L. C, and H. C, respectively. Treatment with Fe_3_O_4_/SA nanoparticles significantly increased germination percentage to 99% and 68% at low and high heavy metals concentrations, respectively (Table 2).

**Table 2:** Effect of different concentrations of heavy metals in presence or absence of carbonized rice straw and Fe3O4/SA nanoparticles treatments on germination percentage (%), seedling vigor index (SVI), metal tolerance idex (MTI) and some growth criteria of alfalfa. during seed germination (7-days-old)

Heavy metals	Germination %	SVI	MTI	Plumule length (cm)	Radicle length (cm)	Seedling Fresh Weight (mg)	Seedling Dry Weight (mg)
0	92 ± 2.4 b	800.4 ± 21.2 b	100 ± 2.64 a	5.3 ± 0.11 bc	3.4 ± 0.09 a	3 ± 0.08 c	0.21 ± 0.06 b
L. C	65 ± 1.7 c	343.1 ± 9.1 e	44.1 ± 1.17 c	3.2 ± 0.09 c	1.5 ± 0.04 bc	2.6 ± 0.07 d	0.18 ± 0.005 c
H. C	34 ± 0.89 d	156.0 ± 4.2 f	14.7 ± 0.39 e	2.1 ± 0.05 c	0.5 ± 0.009 c	2.3 ± 0.06 e	0.13 ± 0.003 e
L. C + C.R.S	94 ± 2.5 ab	807.5 ± 21.4 b	58.82 ± 1.55 b	6.5 ± 0.16 a	2 ± 0.05 b	4.3 ± 1 a	0.17 ± 0.005 cd
L. C + Fe_3_O_4_/SA nanoparticles	99 ± 2.6 a	883.2 ± 23.4 a	102.9 ± 2.72 a	5.7 ± 0.14 bc	3.5 ± 0.09 a	3.6 ± 0.09 b	0.23 ± 0.006 a
H. C + C.R.S	69 ± 1.8 c	556.8 ± 14.7 d	38.2 ± 1.01 d	5.1 ± 0.11 b	1.3 ± 0.03 bc	3 ± 0.08 c	0.16 ± 0.005 d
H. C + Fe_3_O_4_/SA nanoparticles	68 ± 1.7 c	620.5 ± 16.4 c	44.1 ± 1.17 c	5.8 ± 0.15 bc	1.5 ± 0.04 bc	2.6 ± 0.07 d	0.17 ± 0.005 cd
LSD at 5%	7	63.7	5.9	2	1.4	0.4	0.02

Data shown in the table represents the mean ±standard error, followed by a small letter; similar letters indicate that means were not significantly at 5%, probability based on Duncan’s test

Seed vigor index, MTI, Plumule and radicle lengths, fresh and dry weight were adversely impacted by increase in heavy metals concentration (Table 2). At high concentrations, radicle length decreased to became 0.5 cm compared to control (3.4 cm). Plumule length decreased to 2.1 cm compared to 5.3 cm for control. The greatest reduction was in SVI, which decreased from 800.4 to 156. Seedling fresh and dry weight significantly decreased as the concentration of heavy metals increased.

**Fig. 7:**
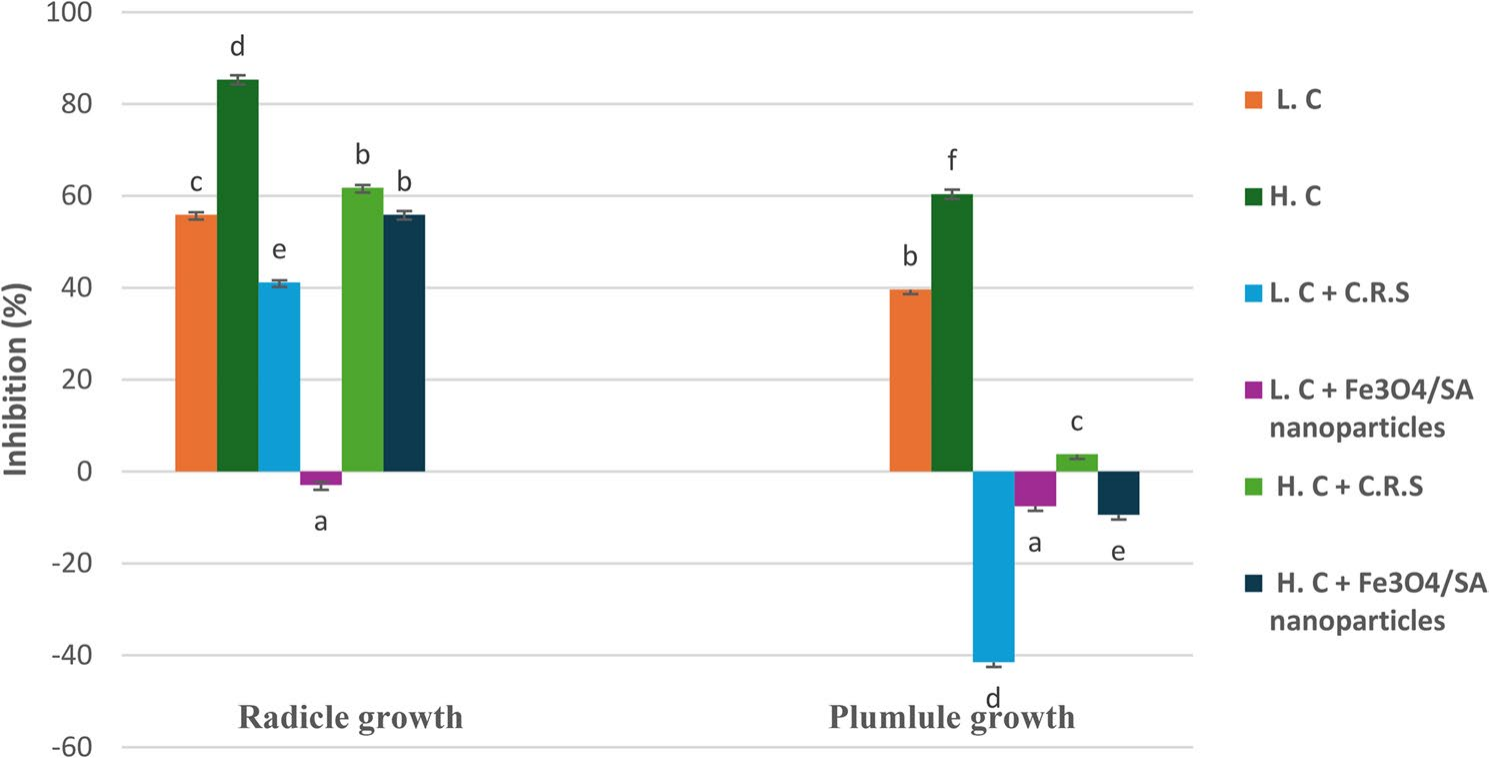
Effect of different concentrations of heavy metals in presence or absence of carbonized rice straw and Fe_3_O_4_/SA nanoparticles treatments on Inhibition (%) of radicle growth, Inhibition (%) of plumule growth. Different letters (a, b, c, d, e and f) indicate statistical differences at 5% probability according to Duncan’s test. Error bars are standard errors of the mean

The inhibition percentage for both r$\:\mathrm{a}\mathrm{d}\mathrm{i}\mathrm{c}\mathrm{l}\mathrm{e}$ and plumule increased dramatically at high concentration of heavy metals reaching 85.29% for radicle and 60.37% for plumule (Fig. 7). All assessed growth criteria of alfalfa seedlings considerably increased by the application of carbonized rice straw or Fe_3_O_4_/SA nanoparticles. Fe_3_O_4_/SA treatment exhibited a noticeable significant increase in radicle length to reach 3.5 and 1.5 cm at low and high levels of heavy metals respectively (Table 2), followed by application of carbonized rice straw 2 and 1.3 cm respectively. The highest plumule length was recorded by the application of carbonized rice straw with low levels of heavy metals recording 6.5 cm. Fresh and dry weight of alfalfa germinated seeds was significantly improved by application of Fe_3_O_4_/SA nanoparticles or carbonized rice compared to heavy metals stressed seedlings (Fig. 7). Metal tolerance index demonstrated a significant decline as heavy metal concentration increased, reaching 14.7%, MTI values increased when carbonized rice straw or Fe_3_O_4_/SA nanoparticles were applied; the highest value 102.9%, was obtained with the treatment of L. C with Fe_3_O_4_/SA nanoparticles (Table 2).

### Activity of hydrolytic enzymes

The activity of α-amylase and protease hydrolytic enzymes drastically decreased as the concentration of heavy metals increased (Table 3). Applying carbonized rice straw or Fe_3_O_4_/SA nanoparticles significantly reduced the negative effects of heavy metals and increased enzyme activity to levels above or close to the control value.

**Table 3:** Effect of different concentrations of heavy metals in presence or absence of carbonized rice straw and Fe3O4/SA nanoparticles treatments on total soluble sugars, total soluble protein (mg g^-1^ f.wt) and the activity of some hydrolytic enzymes (amylase and protease) (mg g^-1^ f.wt min^-1^) during seed germination of alfalfa. (7-d-old)

Heavy metals	α-amylase	Protease	Total soluble sugars	Total soluble proteins
0	57.4 ± 1.5 a	0.169 ± 0.004 ab	6.63 ± 0.1 c	73.8 ± 1.9 c
L. C	43.9 ± 1.2 d	0.158 ± 0.004 bc	6.43 ± 0.17 c	65.9 ± 1.7 d
H. C	43.4 ± 1.1 d	0.148 ± 0.003 c	5.66 ± 0.14 d	62.6 ± 1.6 d
L. C + C.R.S	51.1 ± 1.3 c	0.178 ± 0.005 a	7.33 ± 0.19 b	85.0 ± 2.2 b
L. C + Fe3O4/SA nanoparticles	55.9 ± 1.4 ab	0.173 ± 0.005 a	8.90 ± 0.23 a	91.6 ± 2.4 a
H. C + C.R.S	50.7 ± 1.3 c	0.174 ± 0.005 a	6.51 ± 0.17 c	75.5 ± 1.4 c
H. C + Fe3O4/SA nanoparticles	52.4 ± 1.3 bc	0.164± 0.004 ab	7.04 ± 0.22 bc	77.1 ± 2 c
LSD at 5%	4.8	0.013	0.77	6.5

Data shown in the table represents the mean ± standard error, followed by a small letter; similar letters indicate that means were not significantly at 5%, probability based on Duncan’s test

**Fig. 8:**
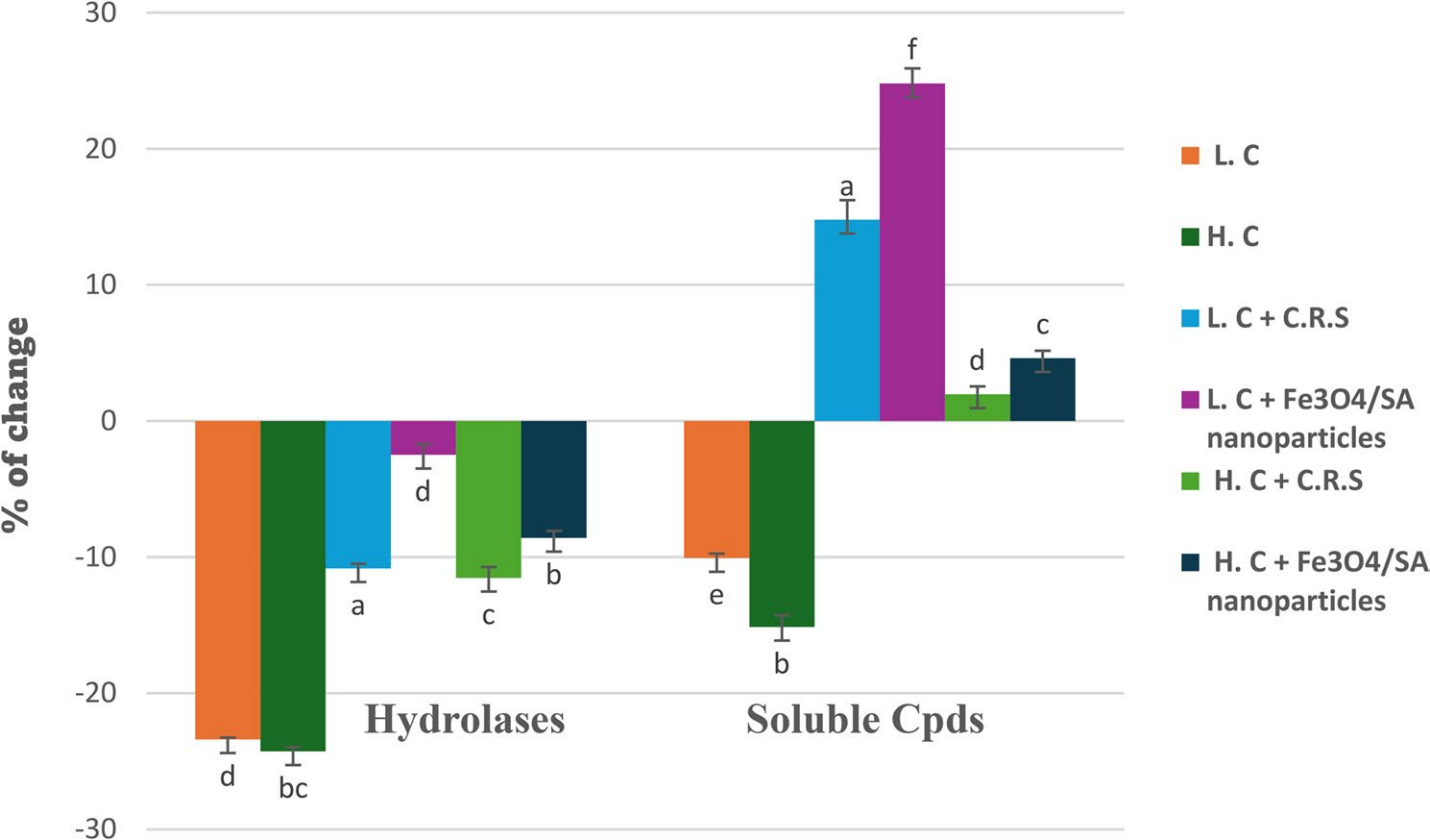
Percentage of change in hydrolases (α-amylase and protease) activity, total soluble compounds (total soluble sugars and proteins) content in response to different concentrations of heavy metals in presence or absence of carbonized rice straw and Fe_3_O_4_/SA nanoparticles treatments. Different letters (a, b, c, d, e and f) indicate statistical differences at 5% probability according to Duncan’s test. Error bars are standard errors of the mean

Hydrolases activity at L. C and H. C of heavy metals decreased by 23.39 and 24.27% respectively compared to corresponding control, while treatment with and Fe_3_O_4_/SA nanoparticles increased hydrolytic enzymes activity decreasing reduction percentage to 10.83 and 11.53% by C.R.S and 2.48 and 8.59% by Fe_3_O_4_/SA nanoparticles at low and high concentrations (Fig. 8).

### Total soluble sugars and proteins contents

Increasing heavy metals concentration resulted in a significant decrease in total soluble sugars and protein content (Table 3). Addition of carbonized rice straw to low and high heavy metals solutions, resulted in increase in total soluble sugars (7.33 and 6.51 mg g⁻¹ f.wt) and total soluble proteins (85 and 75.5 mg g⁻¹ f.wt) compared to significant decrease recorded at L. C and H. C of heavy metals in soluble sugars (6.43 and 5.66 mg g⁻¹ f.wt) and (65.9 and 62.6 mg g⁻¹ f.wt) for soluble protein. (Fig. 8).

### H_2_O_2_ content, Lipid peroxidation levels, and electrolyte leakage

Stress markers of hydrogen peroxide, lipid peroxidation (MDA), and electrolyte leakage, which are indicators of cell membrane damage. At high concentration of heavy metals H_2_O_2_ and MDA content increased to 0.327 µM g⁻¹ f.wt for hydrogen peroxide and 56.89 µ mol g^− 1^ f.wt for lipid peroxidation relative to control. The electrolyte leakage showed the same trend of H_2_O_2_ and MDA content under high concentration of heavy metals that increased from 59.7 to 82.0%, compared to control value (Table 4). Treating Higher concentrations of heavy metal with carbonized rice straw or Fe_3_O_4_/SA nanoparticles reduced the accumulation of H_2_O_2_ to 0.27 and 0.285 µM g⁻¹ f.wt and its destructive effect on the plasma membrane that led to reduction in lipid peroxidation to 32.4 and 35.2 µ mol g^− 1^ f.wt. The electrolyte leakage was considerably reduced to 76.4 and 77.9% after application carbonized rice straw and Fe_3_O_4_/SA nanoparticles treatments.

**Table 4:** Effect of different concentrations of heavy metals in presence or absence of carbonized rice straw and Fe3O4/SA nanoparticles treatments on hydrogen peroxide (H_2_O_2_) content (µM g^-1^ f.wt), Proline (mg g^-1^ f.wt), electrolyte leakage (%), lipid peroxidation (µ mol g^-1^ f.wt) and antioxidants compounds (phenolics, flavonoids (mg g^-1^ f.wt) during seed germination of alfalfa. (7-d-old)

Heavy metals	H_2_O_2_	Lipid peroxidation	Electrolyte leakage	Proline	phenolics	Flavonoids
0	0.19 ± 0.005 e	28.03 ± 0.74 e	59.7 ± 1.57 d	5.8 ± 0.15 c	61.11 ± 1.62 bc	11.13 ± 0.28 b
L. C	0.297 ± 0.008 b	52.06 ± 1.37 b	71.4 ± 1.88 bc	8.1 ± 0.21 b	64.21 ± 1.66 b	12.54 ± 0.32 c
H. C	0.327 ± 0.009 a	56.89 ± 1.50 a	82 ± 2.16 a	13.9 ± 0.36 a	71.13 ± 1.84 a	15.45 ± 0.40 a
L. C + C.R.S	0.20 ± 0.005 e	28.1 ± 0.74 e	65.9 ± 1.74 c	4.7 ± 0.12 e	49.76 ± 1.29 e	11.33 ± 0.29 d
L. C + Fe_3_O_4_/SA nanoparticles	0.227 ± 0.007 d	30.6 ± 0.80 de	67.1 ± 1.82 c	5.5 ± 0.14 cd	55.48 ± 1.44 d	10.83 ± 0.27 d
H. C + C.R.S	0.27 ± 0.008 c	32.4 ± 0.86 cd	76.4 ± 2.02 ab	4.9 ± 0.12 de	60.70 ± 1.57 bc	14.75 ± 0.38 ab
H. C + Fe_3_O_4_/SA nanoparticles	0.285 ± 0.008 bc	35.2 ± 0.93 c	77.9 ± 2.06 a	6.0 ± 0.15 c	58.59 ± 1.52 cd	14.24 ± 0.37 b
LSD at 5%	0.024	4.29	6.2	0.9	5.22	1.2

Data shown in the table represents the mean ± standard error, followed by a small letter; similar letters indicate that means were not significantly at 5%, probability based on Duncan’s test

### Proline content

**Fig. 9:**
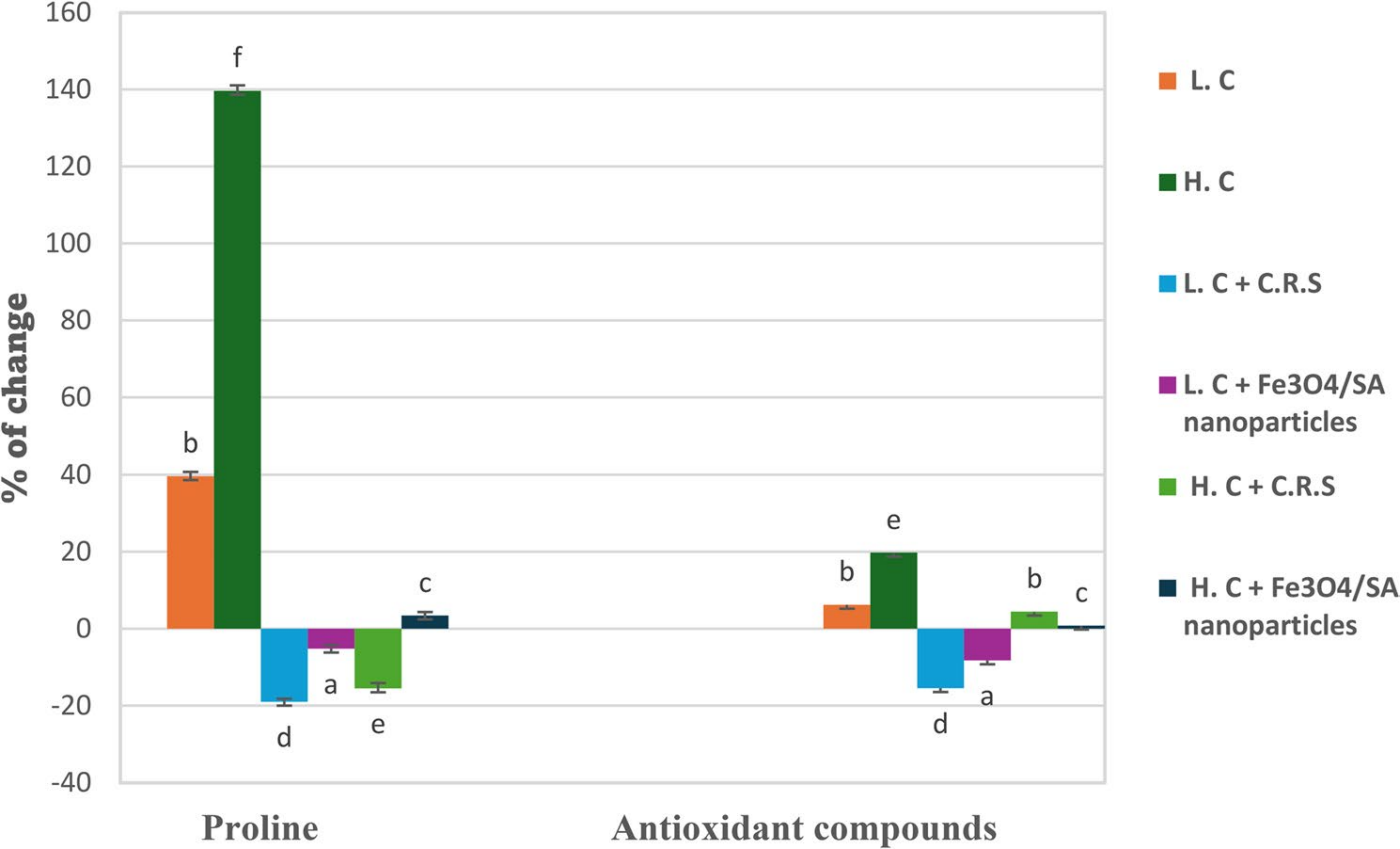
Percentage of changes in proline, antioxidants compounds content (phenolics and flavonoids) in response to different concentrations of heavy metals in presence or absence of carbonized rice straw and Fe_3_O_4_/SA nanoparticles treatments. Different letters (a, b, c, d, e and f) indicate statistical differences at 5% probability according to Duncan’s test. Error bars are standard errors of the mean

The accumulation of proline is a common stress response that at high concentration of heavy metals, the proline content increased to 13.9 mg g⁻¹ f.wt, by 139.65% compared to control value 5.8 mg g⁻¹ f.wt (Table 4; Fig. 9). By treatment with carbonized rice straw and Fe_3_O_4_/SA nanoparticles, the accumulation of proline content decreased, and the highest reduction was recorded at the treatment L. C + C.R.S (4.7 mg g⁻¹ f.wt by 41.97%).

### Phenolics and flavonoids content

Higher concentrations of heavy metals led to increase in nonenzymatic antioxidant compounds (phenolics and flavonoids), by 6.24 and 19.78%, in comparison to the control values (Fig. 9). Treating higher concentrations of heavy metals with carbonized rice straw or Fe_3_O_4_/SA nanoparticles increased the content of phenolic compounds that reached to 60.70 and 58.59 mg g^− 1^ f.wt, compared to 71.13 mg g^− 1^ f.wt at high concentration of heavy metals, while the content of flavonoid compounds became 14.75 and 14.24 mg g^− 1^ f.wt, compared to 15.45 mg g^− 1^ f.wt at high concentration of heavy metals (Table 4). The percentage of changes indicated that the antioxidant compounds (phenolics, flavonoids), were positively affected by carbonized rice straw or Fe_3_O_4_/SA nanoparticles that revealed negative values especially at low levels of heavy metal solution.

### Principle component analysis

**Fig. 10:**
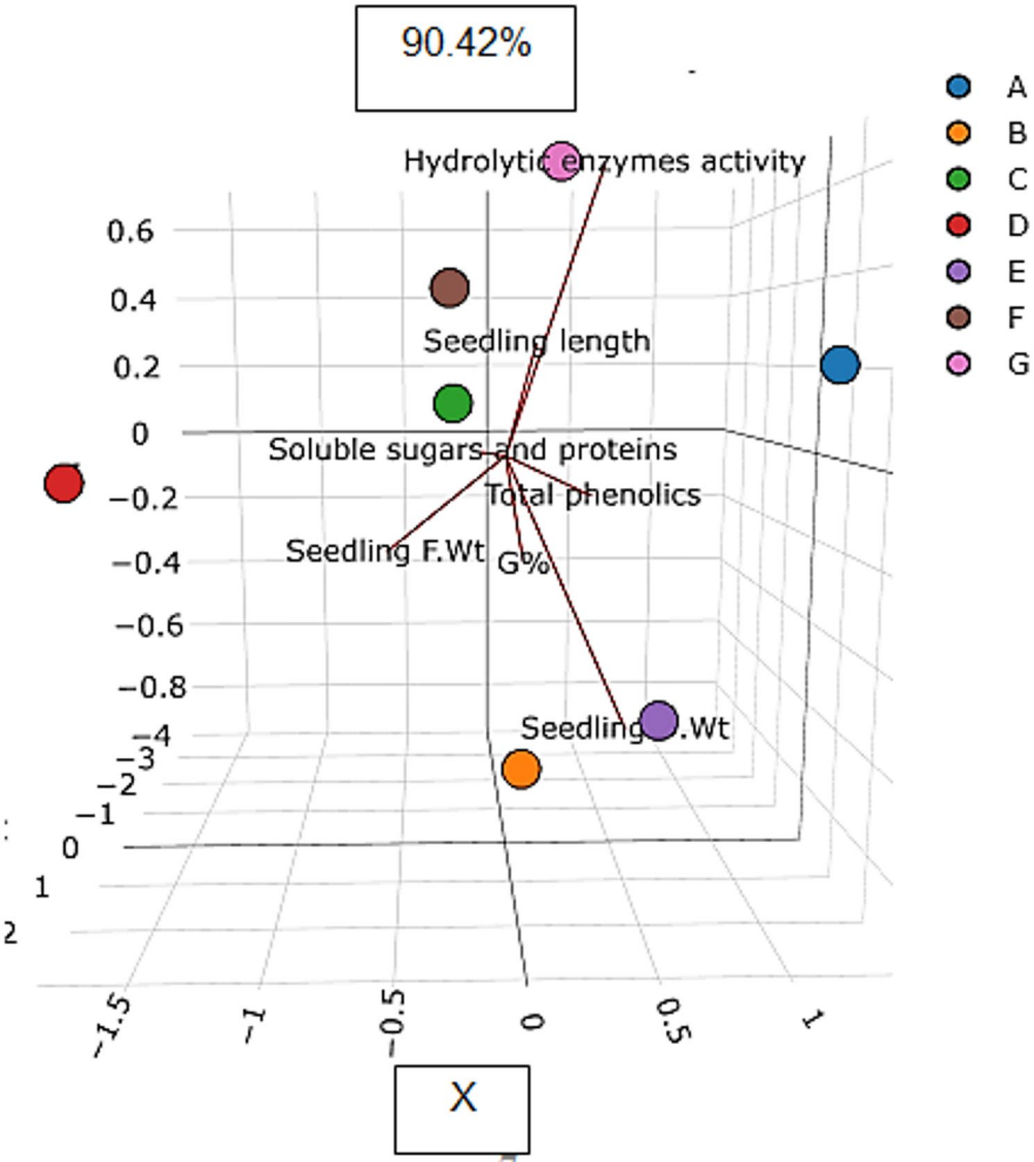
Principal component analysis between treatments and measured parameters and metabolic activities. A = 0; B = L. C; C = H. C; D = L. C + C.R.S; E = L. C + Fe_3_O_4_/SA nanoparticles; F = H. C + C.R.S; G = H. C + Fe_3_O_4_/SA nanoparticles

The 3D PCA biplot (Fig. 10) demonstrated a clear separation among the treatments based on the measured growth and biochemical metabolites and markers. The first principal components effectively explained the variation in hydrolytic enzymes activity, seedling length, soluble sugars, proteins, total phenolics, germination percentage (G%), and seedling fresh and dry weight. Hydrolytic enzymes activity showed a strong positive association with seedling length. Fresh weight and dry weight appeared closely related, suggesting a strong positive correlation between these two growth parameters. In contrast, total phenolics exhibited an opposite directional trend compared to some growth traits, indicating a potential negative association. Treatments positioned in the direction of specific vectors exhibited higher values of the corresponding traits.

## Discussion

In the present study results revealed the potential of C.R.S biochar and Fe_3_O_4_/SA in removal of lead, cadmium, and nickel ions from irrigation water due to their large surface area and high adsorptive power. Biochar is an efficient and eco-friendly catalyst for removing a variety of pollutants, including heavy metals [48], due to beneficial features including cation exchange capacity, porosity, and high surface area and strong adsorptive property [49]. Applying biochar increases pH, soil organic matter content, and cation exchange capacity while also enhancing nutrient retention and reducing the toxicity, mobility, and bioavailability of metals, metalloids, and radionuclides [50]. The composition of biochar makes it also a source of elements including N, P, K, Ca, and Mg, according to Saleem et al. [51]. Through ion exchange, precipitation, and surface chelation, biochar reduced the absorption of Cu, Ni, Zn, Pb, and Cd [52]. The chemical and physical properties of the adsorbent determine how biochar extracts heavy metals from an aqueous solution. The formation of micropores in the pore structure, large specific surface area of the adsorbent, and the weak Vander wall force of attraction between the adsorbent and the heavy metals all contribute to physical adsorption [53]. Biochar, which usually has a negative charge on its valency shell due to the disassociation of oxygen-containing functional groups, like carboxyl (R–COOH) and hydroxyl (–OH). These groups help reduce Cr (VI) to Cr (III) and adsorb metal ions such as Cr (III) and Al (III), especially at low pH, this makes biochar effective in removing pollutants like Cu (II), Ni (II), Pb (II), Zn (II), Cd (II), and Hg (II) [54].

In the present study Fe_3_O_4_/SA nanoparticles UV-Vis. spectroscopy showed a sharp peak at 273 nm. The same result reported by Mohammed et al. [55] who reported a characteristic peak for Fe_3_O_4_ nanoparticles between 259 and 383 nm. The FTIR spectrums showed several functional group peaks that confirmed the surface functionalization of the nanoparticles. According to Khan and Rafiquee [56], the FT-IR spectra of the Fe_3_O_4_ nanoparticles show the presence of Fe-O characteristic vibration bands and absorption bands at 561 cm⁻¹, which represent the unique features. Additionally, peaks that observed at 1632 cm⁻¹ and 3424 cm⁻¹ are associated with vibrational modes related to hydroxyl groups. The shift of the carboxylic band from 1657 cm⁻¹ in pure salicylic acid to 1557 cm⁻¹ in Fe_3_O_4_/salicylic acid nanoparticles may attributed to a reduced C = O force constant caused by surface interactions and anionic conjugation of the –O–C = O group. Additionally, the retention of characteristic peaks from both salicylic acid and iron oxide nanoparticles suggests the formation of a complex or interaction between the two materials [57].

Iron, particularly in nanoparticle form, undergoes surface oxidation and hydrolysis in the presence of water and dissolved oxygen, forming compounds such as iron oxyhydroxide. Surface iron atoms act as Lewis’s acids, interacting with electron-donating species. The amphoteric hydroxyl groups can react with both acids and bases. Due to their physicochemical properties and chemisorption capacity, magnetic particles can effectively remove heavy metals from wastewater through ion adsorption. In aqueous solutions, metal ions either remain stable or hydrolyze into various hydroxyl complexes [58]. Heavy metal retention on iron oxides primarily caused by surface complexation, surface precipitation, and structural incorporation, which are highly dependent on metal ion species, reacting conditions (pH, heavy metal concentration, ionic strength), and chemical processes [59].

Toxic heavy metal pollution of water is a major contributor to this issue, which can lead to disease transmission, ecosystem destruction, and hazardous drinking water [60]. Lead, arsenic, cadmium, nickel, copper, zinc, chromium, and mercury are among the major toxic and hazardous heavy metals found in environmental contamination, according to the WHO.

Our research showed that alfalfa seeds exposed to different concentrations of heavy metals (cadmium, lead, and nickel), showed a significant decrease in the percentage of germination and overall measured growth criteria. The combination of heavy metals in the media, whether at low or high concentrations, can disrupt the uptake of vital nutrients, resulting in deficiencies and imbalances that impact the growth and development of plants. The reduction in growth is attributed to the direct or indirect inhibitory effects of heavy metals on hydrolytic enzymes. Heavy metals can interfere with enzyme structure and function, leading to a significant decrease in the activity of hydrolases such as amylases and proteases. As a result, the breakdown of stored reserves is impaired, causing a reduction in the availability of soluble sugars and proteins that are essential for embryo development. This deficiency limits the energy supply and metabolic processes required for normal growth, leading to suppressed seed germination and reduced seedling development. Our results are similar to Uslu et al. [61], who showed that increased concentrations of heavy metals in irrigation water decreased the rate of seed germination and had a negative effect on the early growth of seedlings of various field crops. As reported by Hafeez et al. [62], heavy metals have a negative effect on the physiology and morphology of seeds, which hinders the germination process and the early growth of plant seedlings. Although they are not necessary for plants, the hazardous trace metals cadmium and lead can readily be absorbed by roots, built up in different organs, and harm plants irreversibly. The connection between the detrimental effects of toxic metals on plant ionic homeostasis and the suppression of plant growth under toxic metal stress, including that caused by Cd and Pb reported by [63].

Heavy metal stress also damages cell membranes by causing lipid peroxidation due to dramatic increase in ROS level, which also damages proteins and nucleic acids in plant cells. Plant growth and yield will eventually be hampered if these radicals are not promptly removed [62]. Damage to the cell membrane disrupts its integrity, resulting in increased permeability and uncontrolled leakage of ions [64]. Seedling vigor index and metal tolerance index decreased, while phytol-toxicity (%) increased with increasing Cd concentration to become 100% at 5 mM CdCl_2_. Cd-stress increased malondialdehyde, hydrogen peroxide and proline content, whereas the total soluble sugars, total soluble proteins, DNA and RNA content decreased [65]. In certain crops, cadmium poisoning also decreases nutrient and water intake, which inhibits the morphological, physiological, and functional development and function of plants and interferes with plant metabolism [66]. Gene expression, enzyme activity, and metabolite levels in alfalfa were examined in relation to Cd stress [67], it was shown that 1 mM Cd increased electrolyte leakage and decreased total soluble protein levels, while H_2_O_2_ content increased as an indicator of oxidative stress. According to Xiao-Yu et al. [68], the reduction in biomass and growth inhibition brought on by Pb exposure may be explained by Pb’s suppression of nutrient absorption, which degrades plants’ photosynthetic activity and impairs cell membrane permeability. Increased Pb concentrations in the soil can cause a range of reactive oxygen species (ROS) to be produced by plant cells. Plant growth is negatively impacted by the significant oxidative damage caused by this increase in ROS [69]. By disrupting the enzyme-substrate complex, denaturing the enzyme protein, and interfering with the active sites, heavy metals can reduce the activity of enzymes [70].

One of the crucial ways that plants react to heavy metal stress is by accumulation of osmolytes including proline in which accumulation in high concentration is considered a defense mechanism against ROS buildup, osmo-protectant and osmo-regulator to maintain water uptake and preserve water content in plant tissue. Plant growth, development, metabolism, and nutrient cycling are all impacted by protein, and changes in protein concentration can reveal how resilient a plant is to stress [71]. Two enzymes that catalyze proline synthesis from glutamate in the cytoplasm or chloroplast, two enzymes that catalyze proline catabolism back to glutamate in the mitochondria, and an additional route of proline synthesis via ornithine comprise the core of proline metabolism [72]. Proline levels are believed to be regulated by the transcriptional upregulation of proline synthesis from glutamate and the downregulation of proline catabolism during stress [73]. The processes involved in proline production and degradation are regulated by several genes. A rate-limiting enzyme called D1-pyrroline-5-carboxylate synthetase (P5CS) transforms glutamate into pyrroline-5-carboxylate (P5C) during the production of proline in higher plants. The second enzyme, D1-pyrroline-5-carboxylate reductase (P5CR), subsequently converts P5C to proline. Abscisic acid (ABA) and nat-siRNAs are involved in the regulation of the P5CS, which is also influenced by alternative splicing and epigenetic control [74].

Our findings demonstrated that the use of carbonized rice straw biochar mitigates the detrimental physiological effects of heavy metal contamination by lowering the toxicity and bioavailability of heavy metals. As reported by Zainul et al. [75], adding rice straw biochar to Cu-contaminated soil enhanced the biomass and growth characteristics of plants. Additionally, it decreased the amounts of malondialdehyde and free proline in plants as well as the activity of antioxidant enzymes, as well as the notable improvement in the chemical characteristics of the soil (pH, electrical conductivity, and cation exchange capacity). Because the antioxidant defense systems were activated, plants treated with 0.75% biochar showed reduced oxidative stress. Osmotic adjustment (proline, soluble sugar, and soluble protein) is widely acknowledged as offering high-energy reactions to sustain cell turgor, which is essential for crop growth, in addition to the scavenging impact of ROS [76]. Jabborova et al. [77] demonstrated that the biochar treatments of maize, alfalfa, and amaranth considerably increased plant height and root morphological features (total root length, root diameter, and root volume. According to the findings of Mu et al. [78]. Potentially toxic element (PTE) stress in maize was successfully reduced by applying rice straw biochar to soils contaminated with Cd, Pb, and Zn, significantly decreased the buildup of PTE in maize shoots. Furthermore, rice straw biochar increased the generation of photosynthetic pigments and enhanced gas exchange efficiency, which improved the photosynthetic apparatus’s performance [79]. Our results demonstrated that Fe_3_O_4_/salicylic acid nanoparticles are highly effective in removing heavy metals from water. This efficiency can be attributed to their large surface area and magnetic properties, which are further enhanced by the presence of salicylic acid. Consistent with our findings, numerous studies have reported the successful application of iron oxide-based materials for heavy metal removal.

The maximum lead ion removal uptake was found to be 93 ± 0.13% at pH 6.0 with 0.4 g of these nanoparticles within 60 min of contact time, indicating that these regenerable iron oxide nanoparticles can be used as nano-adsorbent for the removal of heavy metals from environmental waste due to their high metal uptake capacity [80]. According to Sosun et al. [81], Fe_3_O_4_ nanoparticles have superior adsorption capacities at fundamental circumstances, with higher adsorption values noted for Ni²⁺. In contrast to many other techniques used to assess Cd (II) ions in various environmental and industrial wastewater samples, Abdolmohammad-Zadeh and Salimi [28], concluded that magnetic solid phase extraction based on *Fe*_*3*_*O*_*4*_/salicylic acid nano-sorbent offers an environmentally friendly, straightforward, sensitive, quick, and economical method with comparable or better analytical characteristics. Overall, these results confirm that *Fe*_*3*_*O*_*4*_ nanoparticles play a crucial role in mitigating heavy metal-induced oxidative stress and improving plant resilience. *Fe*_*3*_*O*_*4*_-based nanomaterials enhance oxidative resistance and regulate antioxidant systems, leading to reduced reactive oxygen species (ROS) accumulation and improved stress tolerance [82]. Khalifa and Soliman [83] stated that the removal effectiveness of Pb²⁺ ions may be increased by combining seaweed with nanoparticles (such as iron oxide). Seaweed, iron oxide nanoparticles, and their combination were utilized in this work to remove up to 50.0 mg L⁻¹of lead from contaminated water. With a removal effectiveness of 91%, the designed composite outperformed the other sorbents in the study, which were all successful in lead sorption, according to the results. Applications of nanoparticles have been shown to alter the ultrastructure of leaf organelles, activate antioxidant mechanisms, and improve cellular water balance as demonstrated by [84]. According to He et al. [85], Fe_3_O_4_ nanoparticles protected plant roots from Cd-induced structural damage and cell death by enhancing plant height (23–31%) and biomass (17–24%), while reducing Cd accumulation (25–37%) through adsorption and root immobilization. This improvement was associated with increased nutrient uptake, enhanced stomatal conductance, and higher photosynthetic activity. In addition, Fe_3_O_4_ nanoparticles promoted Cd binding to the cell wall and its sequestration in vacuoles, thereby limiting its entry into organelles and reducing oxidative stress and lipid peroxidation. Under arsenic stress, iron oxide nanoparticles improve plant growth, photosynthesis, membrane integrity, and antioxidant balance. This enhancement is closely associated with regulated carbohydrate metabolism, including increased accumulation of soluble sugars and starch and the activation of key sucrose-metabolizing enzymes. Furthermore, nanoparticle application has been shown to stimulate secondary metabolism, leading to higher accumulation of phenolics and flavonoid compounds, which contribute to stress tolerance by stabilizing cellular structures and reducing oxidative damage [86]. This suggests that these secondary metabolites support improvement of physiological functions and resilience under stress by stabilizing cellular structures and membranes.

The application of *Fe*_*3*_*O*_*4*_/salicylic acid nanoparticles and C.R.S biochar enhanced the antioxidant defense system in alfalfa plant, thereby accelerating the scavenging of reactive oxygen species (ROS). This protective effect reduces lipid peroxidation and malondialdehyde byproduct accumulation, maintains membrane integrity, and regulates cell permeability and ion leakage. Additionally, phenolic and flavonoid compounds act as potent non-enzymatic antioxidants and serve as substrates for certain antioxidant enzymes.

Iron nanoparticles application has the potential to improve bell pepper plants’ resistance to arsenic by boosting growth, glucose metabolism, and secondary metabolite levels, according to Shahzad et al. [86]. Phenolic molecules in plants are essential for adapting to abiotic stressors. The plant accumulates these phenolics to survive under harsh circumstances [87]. Esters, flavonoids, lignin, and tannins are examples of phenolic molecules that function as antioxidants and protect plant cells from various abiotic stressors [88]. The primary accumulator compounds under heavy metal stress are plant phenols. Through their carboxyl and hydroxyl groups, phenolic substances have a propensity to chelate hazardous metals [89].

Cells contain a variety of non-enzymatically acting antioxidant molecules in addition to this cooperative array of enzyme-based antioxidant defense mechanisms [90]. Due to their widespread prevalence in edible plants and increased antioxidant potential, phenolics, especially flavonoids, have received a lot of interest from academia and industry [91]. By interacting with a range of free radicals, phenolic compounds (PCs) function as antioxidants through hydrogen atom transfer, single electron transfer neutralizing reactive oxygen species through hydrogen atom and electron donation, and transition metal chelation [92].

The 3D PCA biplot provides a comprehensive visualization of the relationships among treatments and the measured physiological and biochemical parameters, allowing for clearer interpretation of the overall treatment effects. A strong positive association between hydrolytic enzyme activity, seedling length and biomass accumulation suggests that enhanced enzymatic activity plays a crucial role in promoting seed germination and early seedling development. This can be explained by the role of hydrolytic enzymes in mobilizing stored reserves, thereby supplying energy and essential metabolites required for growth and promoting water uptake and cellular expansion also enhance structural biomass formation. On the other hand, the opposite orientation of total phenolics relative to several growth traits indicates a negative association. This suggests that increased phenolic content may be linked to stress conditions, as phenolic compounds are often synthesized as part of plant defense mechanisms.

## Conclusion

This research investigates the use of carbonized rice straw and iron oxide salicylic acid nanoparticles as affordable, efficient tools for removing heavy metals from irrigation water. By achieving high removal efficiency, these sustainable techniques promote healthier alfalfa development in contaminated environments. Elevated levels of lead, cadmium, and nickel typically hinder the germination and growth of alfalfa by inducing oxidative stress and disrupting metabolic processes. The study demonstrates that these adsorbents significantly reduce metal toxicity, allowing plants to maintain higher levels of soluble sugars and proteins through restoring hydrolytic enzymes activity which are critical for seedling nutrition. This research highlights that utilizing these agricultural and synthetic by-products offers a sustainable solution for large-scale wastewater treatment, enhancing both water quality and agricultural productivity in metal-stressed environments.

### Recommendations

1. The application of carbonized rice straw and Fe_3_O_4_/SA nanoparticles are promising techniques that are highly effective, cost-efficient, and sustainable adsorbents for removing heavy metals (Pb, Cd, Ni) from water.
2. These materials are suitable for large-scale wastewater treatment that improve water quality that achieve maximum removal to pb and cd ions and decreased Ni to exceptionally low concentration.
3. Water treated with carbonized rice straw or Fe_3_O_4_/SA nanoparticles can be effectively utilized for agricultural irrigation. These treatment methods remove heavy metals and contaminants, thereby preserving soil physicochemical properties and promoting healthy plant growth and development.

## Data Availability

Authors can confirm that all relevant data are included in the given article.

## Abbreviations

Fe_3_O_4_/SA nanoparticles: Iron oxide salicylic acid nanoparticles
C.R.S: Carbonized rice straw
L. C: Low concentration of heavy metal solution
H. C: High concentrations of heavy metal solution
EC: electrolytic conductivity
ANOVA: analysis of variance
LSD: least significant difference
